# The radiographic relationship between the cortical overlap view (COV) and the tip of the greater trochanter

**DOI:** 10.1038/s41598-021-97951-8

**Published:** 2021-09-15

**Authors:** Bjorn-Christian Link, Nicole M. van Veelen, Katja Boernert, Piyabuth Kittithamvongs, Frank J.P. Beeres, Hans H. de Boer, Filippo Migliorini, Sven Nebelung, Matthias Knobe, Steffen Ruchholtz, Reto Babst, Chittawee Jiamton

**Affiliations:** 1 Department of Orthopedic and Trauma Surgery, Cantonal Hospital Lucerne, Lucerne, Switzerland; 2 Institute of Orthopedics, Lerdsin Hospital, Bangkok, Thailand; 3 Department of Pathology, Amsterdam UMC, Amsterdam, The Netherlands; 4 Department of Orthopedic, Trauma and Reconstructive Surgery, RWTH University Hospital, Aachen, Germany; 5 Department of Diagnostic and Interventional Radiology, RWTH University Hospital, Aachen, Germany; 6 Center for Orthopedics and Trauma Surgery, University Hospital Giessen and Marburg, Marburg, Germany; 7 Department of Health Sciences and Medicine, University of Lucerne, Lucerne, Switzerland

**Keywords:** Anatomy, Medical research

## Abstract

For proximal femoral nailing, choosing the proper entry point with the aid of C-arm imaging is crucial. Therefore, obtaining accurate radiological views that facilitate sound identification of the tip of the greater trochanter (GT) is of utmost importance. The aim of this study was to define a radiological view characterised by reproducible radiographic landmarks which will allow the reliable identification of the tip of the GT in the anteroposterior view. Anatomical and radiographic features of 16 cadaveric femurs were analysed. The cortical overlap view (COV), characterised by the radiological overlap of the density line of the piriform fossa and the intertrochanteric crest, was identified. It marks the rotation of the proximal femur at which the GT can be accurately identified and used to determine the desired entry point for a proximal femoral nail. Trainees and fully qualified orthopedic trauma surgeons were asked to identify the correct COV in radiological imaging series. Mean internal rotation of the femur to achieve a COV was 17.5° (range 12.8°–21.8°). In the COV the tip of the GT is the highest visible point and the mean distance from the cortical overlap line to the tip of the GT is 4.45 mm. Intra- and inter-rater reliability was high with ICC(2,k) = 0.932 and ICC(2,k) = 0.987 respectively. Trainees achieved higher rates of correct COV identification than specialists. There was no significant correlation between the internal rotation of the femur to achieve the COV and femoral antetorsion. In conclusion, the COV is a highly reproducible radiological view that is characterised by radiographic landmarks easy to recognise. It allows for accurate identification of the tip of the GT, which can be used by the surgeon as a reference to determine the desired entry point for an intramedullary nail.

## Introduction

Trochanteric fractures are common injuries in the aging population, resulting in high morbidity and mortality. The aims of surgical treatment are pain control and early mobilization which facilitate the reintegration into the patient’s social environment with the best possible quality of life. Proximal femoral nailing is reported to achieve satisfactory outcomes for these aims^1,2^ and is the implant of choice in many regions^3^. Complications, however, have a devastating effect on these usually frail patients. Fixation failure occurs in 4–13%^2,4^. Risk factors for mechanical failure include fracture mal-reduction, an increased tip-apex distance (TAD), a suboptimal position of the screw or blade within the femoral head, an improper nail-shaft-axis and varisation of the neck-shaft angle^5–7^ The entry point of the nail has an immense influence on all of these risk factors^8^.

Several studies have described the tip of the greater trochanter (GT) as the standard entry point for trochanteric nails^9^. Further studies defined the optimal entry point either medial^10,11^ or lateral to the tip of GT^12,13^. Differences in the design of the various nail systems as well as the fracture pattern influence the advocated entry point, however, even with identical nails divergent recommendations have been published^8^. Despite the variety of recommendations for the nail entry point, the reference point they all have in common is the tip of the GT. Variations in the morphology of the GT further attribute to difficulties in finding the optimal entry point^14,15^.

Typically, intramedullary nailing of the proximal femur is performed by means of minimally-invasive techniques, precluding direct visualisation of anatomic landmarks. Hence, when choosing the entry point of the nail, the surgeon must rely solely on radiographic imaging. Knowledge of the radiological anatomy of the proximal femur is therefore imperative.

As described, the tip of the GT is of utmost importance as a reference for the nail’s entry point. Yet, visualisation of the GT is strongly dependent on the viewing angle of the proximal femur. The trochanteric tip is radiologically visualised in the antero-posterior (AP) beam path. Acquiring standardized ap views is, however, often inhibited by the rotation of the femur required for fracture reduction. Therefore, the primary aim of this cadaveric study was to identify accurate and reproducible radiographic landmarks to facilitate the accurate determination of the tip of the GT.

## Research methodology

**Figure 1:**
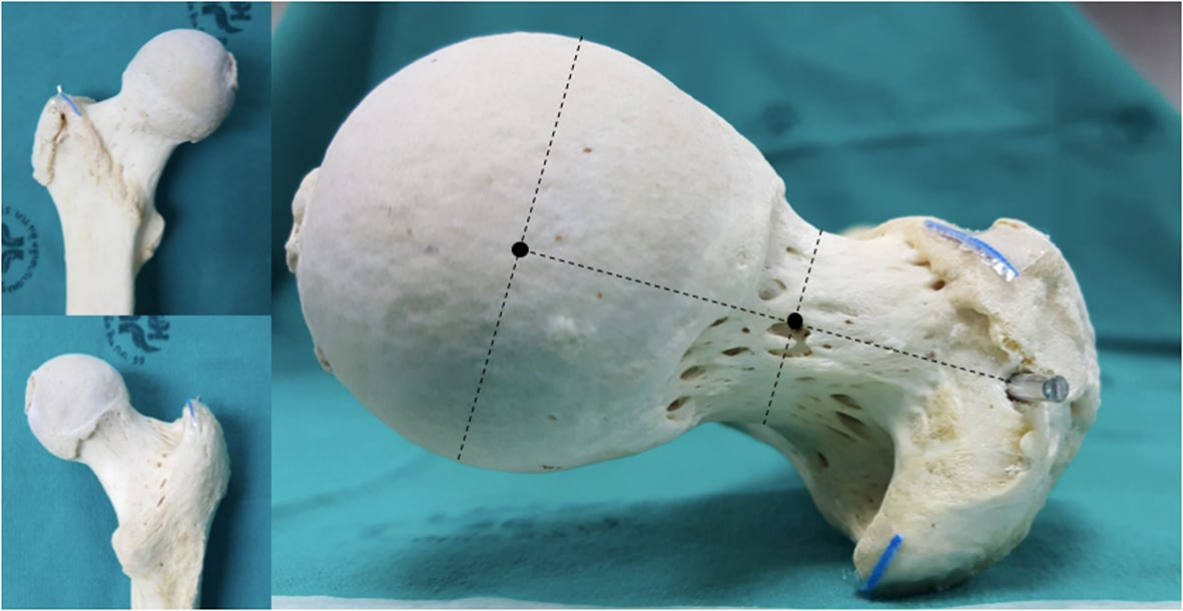
Marking the anatomical landmarks. A wire was inserted into the tip of greater trochanter, which was defined as the intersection between the line connecting the centre of the femoral head with the centre of the femoral neck on axial view and the most proximal aspect along the greater trochanteric crest on the anteroposterior view. The blue lines are markers attached to the antero- and postero-superior borders of the greater trochanter.

Sixteen randomly chosen dried cadaveric femora were used; all of which were obtained via approved anatomical body donation programmes. The protocol complied with the Human Research Act and the guidelines of the Swiss Association of Research Ethics Committees. Institutional review boards of both Lerdsin Hospital and the Lucerne Cantonal Hospital approved the experimental protocol. Informed consent was obtained from the cadaver donors or their next-of-kin by anatomical institues in Thailand and the Netherlands, respectively. Eight femora were obtained from an Asian and eight from a Caucasian population. All bones were confirmed to be free of any history of lower extremity trauma or disease. The examination setup was standardized by positioning the bones in a neutral position which will be defined later in the text. A Kirschner wire was inserted into the tip of the GT, which we defined as the intersection between the line connecting the centre of the femoral head to the centre of the femoral neck on an axial view and the most proximal aspect along the greater trochanteric crest on the ap view (Fig. 1). This intersection was chosen as the greater trochanter is typically a massif with multiple tips rather than a structure with one single tip. Depending on the angle chosen for the ap beam path when visualising the proximal femur different tips may be interpreted as the true tip of the GT. In the axial view the nail entry point should ideally lie in the plan connecting the center of the femoral head and neck to allow for optimal positioning of the cephalic device, irrespective of the chosen implant. Therefore, defining the most proximal aspect of the trochanteric crest in the ap view that corresponds with an optimal axial positioning of the entry point should be beneficial.

A radiopaque marker was attached to the antero-superior and postero-superior border of the greater trochanter to identify the profile of the greater trochanter in the images obtained by C-arm.

**Figure 2:**
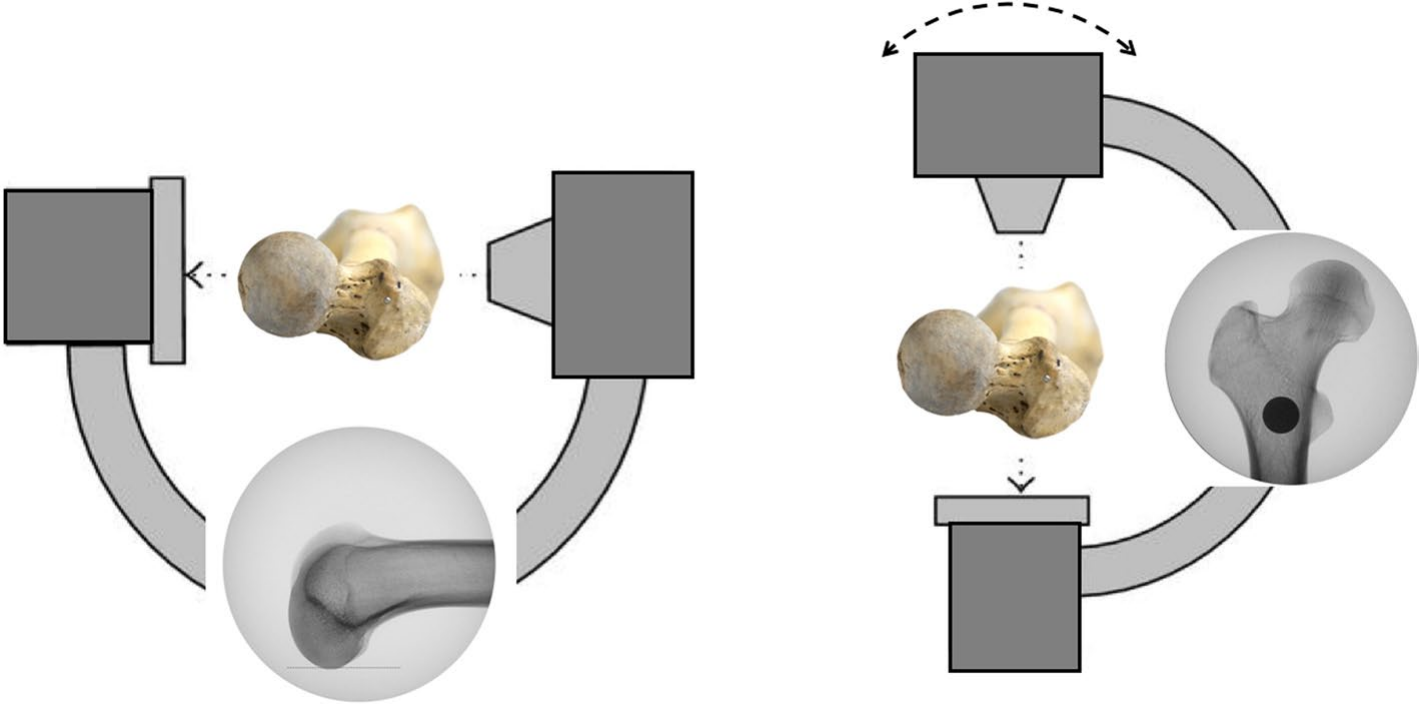
Positioning of the femur and c-arm. Lateral position of the c-arm to determine the neutral position of the femur with perfectly overlapping femoral condyles. The c-arm was moved proximally and into a vertical projection. It was then gradually rotated while obtaining images in 5° intervals from 30° internal rotation to 30° external rotation.

**Figure 3:**
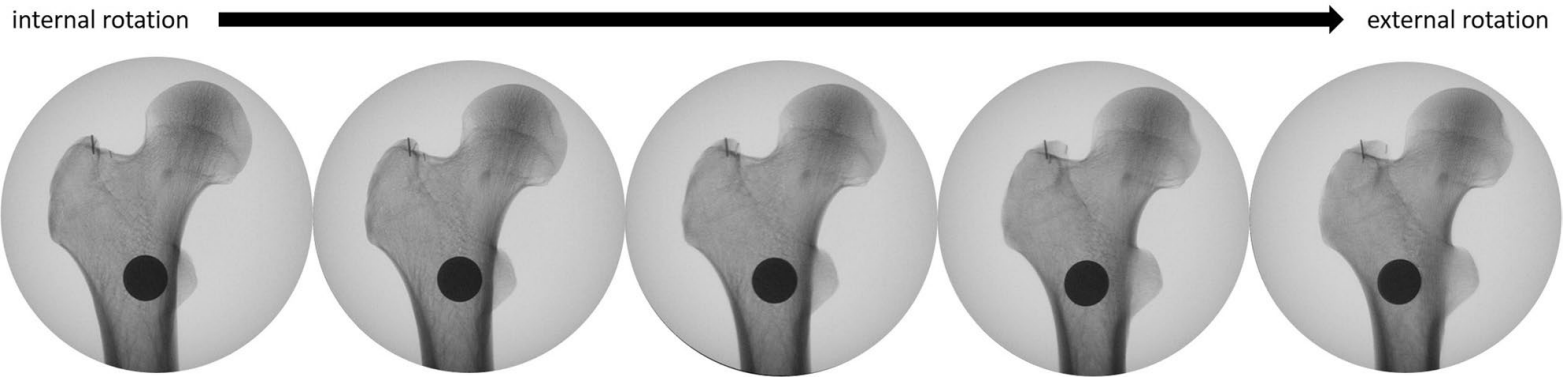
Radiological image sets. Images were attained at various angles as the c-arm was rotated in 5° intervals from internal to external rotation.

In the following step, the neutral position of the femur was determined. The C-arm was positioned in the lateral projection with the beam parallel to the ground and perpendicular to the femoral condyles to obtain a lateral view of the distal femur. The bone was manipulated until the posterior femoral condyles overlapped perfectly and were parallel to the floor. The femur was fixed in this position. The C-arm was then moved proximally parallel to the bone and rotated to the ap projection of the proximal femur (Fig. 2). The C-arm was gradually rotated in 5° intervals from 30° internal to 30° external rotation (Fig. 3). Images of the different viewing angles were saved. Positive viewing angles represent internal rotation, negative viewing angles correspond to external rotation of the femur.

Afterwards, all markers were removed while keeping the bone in the same position. The same procedure as described above with 5° steps of rotation was performed in order to obtain identical images free of markers. Images were stored.

**Figure 4:**
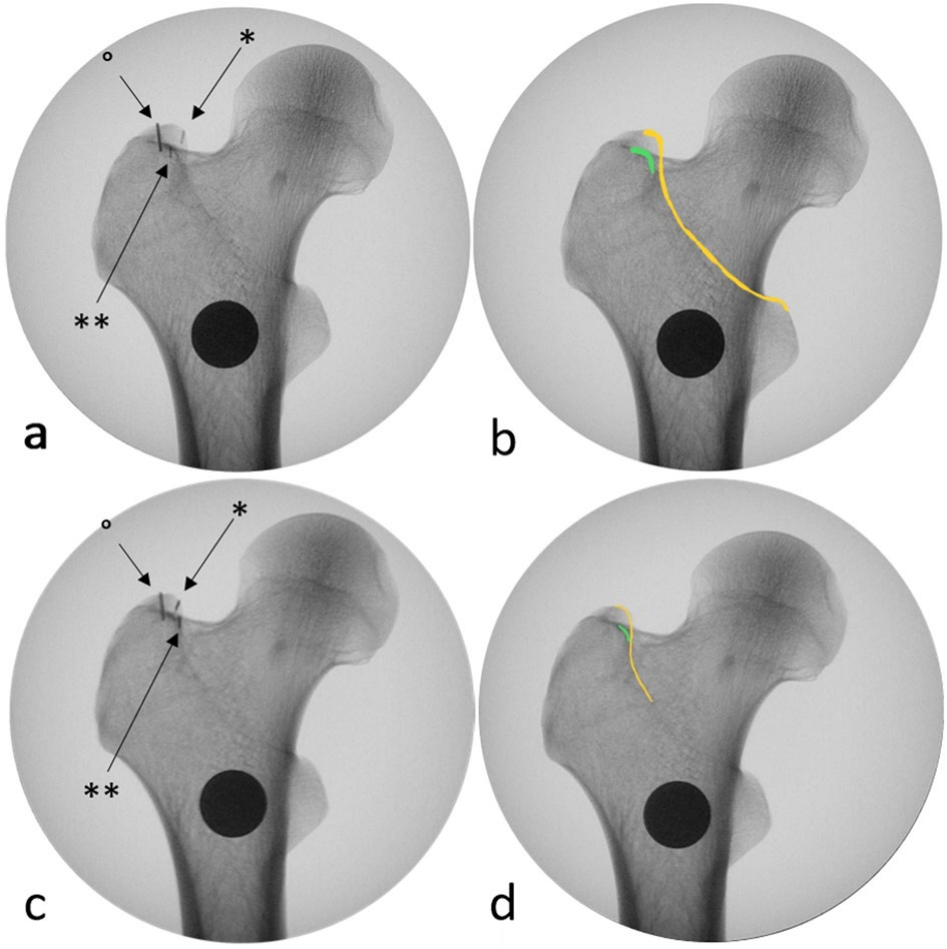
Defining the radiological landmarks. (**a**,**b**) and (**c**,**d**) are each pairs of an identical radiological view of the proximal femur. (**c**,**d**) both show the COV while (**a**,**b**) show an ap view, which deviates from the COV by 10° external rotation. (**a**) xray image with markers indicating the postero-superior border of the GT (*) and the anterio-superior border of the GT (**) as well as the tip of the GT (°). (**b**) the postero-superior border of the GT is the extension of the intertrochanteric crest (yellow). A density line of the piriform fossa is easily recognized (green). (**c**,**d**) the antero- and postero-superior borders of the GT overlap in the COV, this coincides with an overlapping of the easily recognizable intertrochanteric crest and density line of the piriform fossa.

Dependent on the rotation of the C-Arm the antero-superior border, which is often hard to recognize, and postero-superior border of the GT overlap. This coincides with an overlapping of the density line of the piriform fossa and the intertrochanteric crest, which are both easily recognized in c-arm images. (Fig. 4) We named this line the cortical overlap line. The view in which the cortical overlap line is visible was defined as the Cortical Overlap View (COV) (Fig. 3). The position of the C-arm, in which the COV was achieved, was noted.

**Figure 5:**
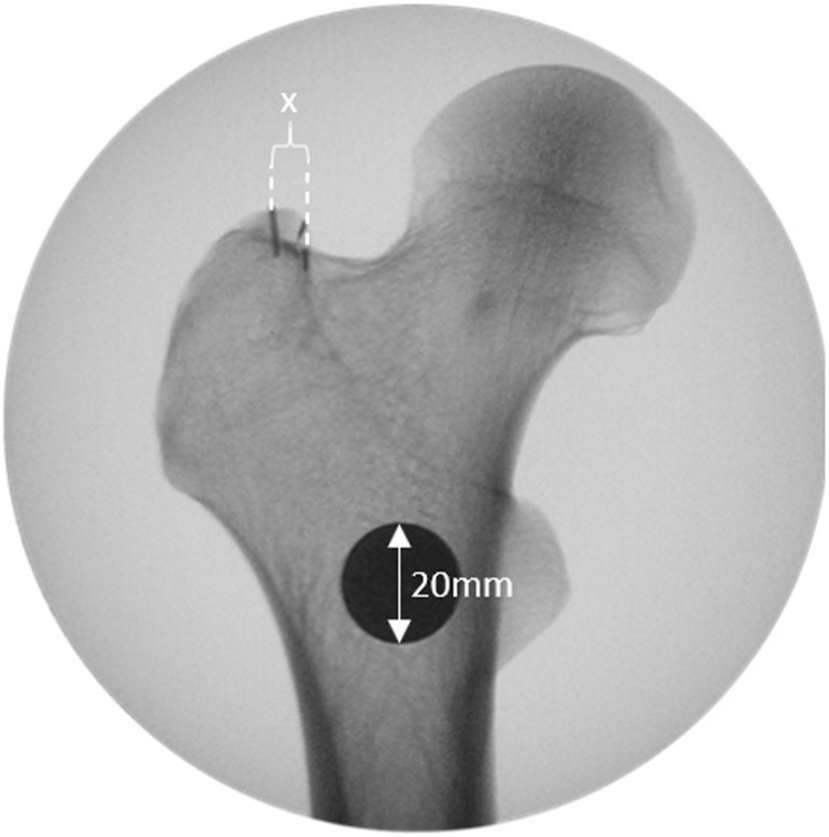
The distance X between the tip of the GT and the cortical overlap line was measured. To calibrate the measurements a coin with a known diameter of 20 mm was place directly on the femur while obtaining the images.

Digital measurements were performed using ImageJ software (ImageJ v1.49, National Institutes of Health, USA). The distance between the cortical overlap line and the tip of the GT (Fig. 5), the distance between the posterior border of the GT to the tip of the GT in the various views and the antetorsion of the proximal femur (defined in the axial plane as the angle between the line defined by the posterior aspect of the distal femoral condyles and a line drawn from the centre of the femoral head to the midline of the femoral neck)^15^ were measured. The type of greater trochanteric overhang according to Grechenig et al. was evaluated in the axial radiograph^16^: group 1, with full access to the entry point; group 2, where the outline of the spine is projected laterally; group 3, where the entry point is partially covered and group 4, where the entry point is completely covered. The length of the femur was defined as the distance in millimetres from the tip of the GT to the most distal part of the lateral femoral condyle.

### Observer variability

To evaluate the inter- and intra-observer reliability of the COV, five orthopaedic surgeons in training and five board certified orthopaedic trauma surgeons were trained and asked to identify the correct COV. The images were all anonymized, randomly ordered within each set of 13 images (30° external rotation to 30° internal rotation in 5° intervals in the same specimen) and then presented to the observers. The observers were asked which imaged showed the cortical overlap view. All images were evaluated by each observer at two different points in time, 4 to 6 weeks apart. The observers were not provided with any feedback between the sessions and the sequence of the images was altered.

### Statistical analysis

The SPSS program (IBM SPSS for windows, version 22) was used for the statistical analysis. Normality of distribution was evaluated by the Shapiro–Wilk test. All descriptive values were reported as mean, standard deviation and 95% confidence interval for parametric data. Correlation between degree to achieve cortical overlap view and antetorsion angle were analysed using Pearson correlation coefficient. Correlation between cortical overlap view and type of greater trochanter overhang were analysed using one-way ANOVA. The correlation coefficient (r) was used to describe the degree of linear relationship between two variables. Strength of linear relationship was classified as very high (r =  > 0.9), high (r = 0.7–0.9), moderately (r = 0.5–0.7), low (r = 0.3–0.5), and little if any correlation (r < 0.3) along with a scatter plot graph. Intra- and Interrater reliability was calculated using the intraclass correlation coefficient (ICC)^17^. Correct COV was defined as COV ± 5°. The ICC (2,K) was selected according to Shrout and Fleiss, because the raters were randomly selected and each subject was measured by the same raters. Additionally, the analysis concentrated on average measures as well as the agreement between raters, including systematic errors of both raters and random residual errors. Interpretation was based on the guidelines proposed by Shrout and Fleiss^18^. An ICC of ≥ 0.7 was considered acceptable, an ICC ≥ 0.8 as good and an ICC ≥ 0.9 as excellent. The level of statistical significance was defined as *p* ≤ 0.05.

## Results

### Cadaveric study

Sixteen femora were included in this study with an average age of 79.06 years (SD: 11.73, 95%CI 72.81–85.31). Nine (56.2%) femora were female and ten (62.5%) femora were right sided. According to Grechenig^16^, eight (50%) femora were type I while four (25%) femora were type II and four (25%) femora were type III. The mean femoral length was 414.75 mm (SD: 26.2, 95%CI 400.79–428.71). Mean antetorsion of the femur was 9.92° (SD: 5.65, 95%CI 6.91–12.93). The mean rotation of the C-arm to achieve COV was + 17.5° (SD: 9.49, 95%CI 12.44–22.55) and the mean distance from the cortical overlap line in the correct COV to the tip of the GT, which is then the highest visible point of the GT, was 4.45 mm (SD: 0.65, 95%CI 4.1–4.79). There was no significant difference between Asian and Caucasian femora for any of the abovementioned variables, except for femoral length (Table 1).

**Table 1:** The demographic data of all specimens (N = 16).

	All (N = 16)	Asian (N = 8)	Caucasian (N = 8)	P value
Age (years)	79.06 ± 11.73	74.88 ± 4.39	83.25 ± 15.34	0.175^a^
**Gender**				0.315^b^
Female	9 (56.2%)	3 (37.5%)	6 (75%)	
Male	7 (43.8%)	5 (62.5%)	2 (25%)	
**Side**				0.119^b^
Left	6 (37.5%)	5 (82.5%)	1 (12.5%)	
Right	10 (62.5%)	3 (37.5%)	7 (87.5%)	
Femoral length (mm)	414.75 ± 26.2	398.88 ± 16	430.63 ± 25.28	0.010^a^
Antetorsion (degree)	9.92 ± 5.65	8.65 ± 4.76	11.19 ± 6.49	0.388^a^
The internal rotation of proximal femur to get COV (degree)	17.5 ± 9.49	13.75 ± 9.54	21.25 ± 8.34	0.116^a^
Distance from COL to Tip of GT (mm)	4.45 ± 0.65	4.31 ± 0.74	4.59 ± 0.57	0.415^a^
**Type of GT**				0.804^b^
1	8 (50%)	5 (62.5%)	3 (37.5%)	
2	4 (25%)	1 (12.5%)	3 (37.5%)	
3	4 (25%)	2 (25.0%)	2 (25.5%)	
4	0	0	0	

*mm* millimeter, *COV* cortical overlap view, *COL* cortical overlap line, *GT* greater trochanter.

^a^Independent sample t-test, ^b^Fisher’s Exact test.

The posterior border of the greater trochanter was positioned medially in relation to the tip of the GT in external rotation and moved laterally with an increasing angle of internal rotation. There was no significant correlation between the internal rotation of the femur to achieve the COV and femoral antetorsion, the type of GT overhang, sex, age or geographic ancestry.

### Observer reliability study

Table 2 shows the individual results including inter-rater reliability of the ten observers. The COV was determined correctly (defined as actual COV ±  5°) in 90.31% of cases. On average, correct determination of COV was achieved in 92.5% for the first test and for the follow-up test in 88.13%. Overall, the doctors in training recognized the correct COV more often than the specialists (board certified orthopaedic trauma surgeons). In the first round the doctors in training identified the correct COV in 95% and the specialists in 90%. In the second round the compliance was 90% and 86.25% respectively. The average for both rounds was therefore 92.5% for the doctors in training and 88.13% for the specialists.

**Table 2:** Individual results of the 10 observers.

	Correct COV, 1st test (%)	Correct COV, 2nd test (%)	Intrarater-reliability	95% CI
Junior1	93.75	87.5	0.962	0.801–0.989
Junior2	100	100	0.980	0.918–0.994
Junior3	93.75	93.75	0.967	0.905–0.988
Junior4	93.75	87.5	0.936	0.818–0.978
Junior5	93.75	81.25	0.913	0.749–0.970
Senior1	81.25	93.75	0.854	0.584–0.949
Senior2	87.5	93.75	0.942	0.838–0.979
Senior3	93.75	87.5	0.985	0.958–0.995
Senior4	100	81.25	0.826	0.491–0.939
Senior5	87.5	75	0.959	0.883–0.986

The intra-class correlation test (ICC) found excellent values for inter-rater reliability with an average of 0.987 (95% CI 0.975–0.994) for both test rounds together. In regard to the first test, agreement was 0.978 (95% CI 0.957–0.991). In the second test the agreement was slightly lower with 0.968 (95% CI 0.937–0.987). Intra-rater reliability was found to be excellent with an average of 0.932 (95% CI 0.826–0.985). Individual results for intra-rater reliability are listed in Table 2.

## Discussion

This cadaveric study was conducted with the aim of defining a reproducible radio-anatomical landmark to assist in identifying the tip of the greater trochanter when using a C-arm. We found the overlap of the intertrochanteric crest with the density line of the piriform fossa to coincide with the overlap of the postero- and antero-superior GT border creating the cortical overlap line and permitting the identification of the tip of the GT. We named this view the cortical overlap view. On average a 17.5° oblique view from lateral to medial (perceived internal rotation of the femur) was necessary to achieve the COV. On average, the tip of the GT was then 4.5 mm lateral to the overlap line. This landmark allowed reliable identification of the GT with excellent intra- and inter-rater reliability.

The proximal femoral nail has gained popularity for the treatment of proximal femur fractures^3^. As these fractures typically affect the elderly, complications such as fixation failure can have a devastating result. The entry point of the nail plays a vital role in the success of nailing. Nevertheless, the definition of the ideal entry point in the coronal plane remains controversial. Not only does it vary depending on fracture pattern, anatomical morphology and the implant used, but divergent recommendations for the same type of nail have been reported^8,11,19^. Furthermore, variations in anatomy can make determining the GT and the potential nail entry point difficult^20^. Grechenig et al*.*^16^ investigated the anatomy of the proximal femur and classified various groups based on the coverage of the piriform fossa (standard entry point for a straight femur nail), supporting the idea of variation in trochanteric anatomy.

In a fracture situation, the fragments can be displaced and rotated, making it difficult to identify the tip of the GT. After positioning the femur in such a way that the fracture is reduced, COV can be achieved by rotating the c-arm until the cortical overlap line is visible. The tip of the GT can then be reliably identified, facilitating the determination of the desired nail entry point.

This new radiological landmark shows excellent inter- and intra-rater reliability, making it a dependable reference for clinical use. Several studies^21,22^ suggested that different levels of clinical experience may have an effect on the inter-observer reliability between two observers. However, we found that the juniors could identify this landmark as well as the seniors.

There are limitations to our study. First, this cadaveric study was conducted in non-fractured femora. The applicability of the COV in a clinical setting, especially if the GT is involved in the fracture, remains to be tested. Second, the number of femoral bones examined was relatively small, potentially causing an underestimation of the anatomical variation which could affect the COV. However, the proportion of the type of greater trochanter overhang in this study was comparable to the previous study by Farhang^14^, permitting the assumption that our sample is representative for the natural variation amongst individuals.

In conclusion, the COV provides reliable landmarks to identify the tip of the GT. This can then be used as a reference when choosing the entry point for an intramedullary nail. The COV showed excellent intra- and inter-rater reliability, though future clinical studies are required to validate the results of this cadaveric study in a clinical setting.

## Data Availability

All data generated or analysed during this study are included in this published article.
